# Green tea polyphenol (epigallocatechin-3-gallate) improves gut dysbiosis and serum bile acids dysregulation in high-fat diet-fed mice

**DOI:** 10.3164/jcbn.18-116

**Published:** 2019-04-06

**Authors:** Chihiro Ushiroda, Yuji Naito, Tomohisa Takagi, Kazuhiko Uchiyama, Katsura Mizushima, Yasuki Higashimura, Zenta Yasukawa, Tsutomu Okubo, Ryo Inoue, Akira Honda, Yasushi Matsuzaki, Yoshito Itoh

**Affiliations:** 1 Molecular Gastroenterology and Hepatology, Graduate School of Medical Science, Kyoto Prefectural University of Medicine, 465 Kajii-cho, Kamigyo-ku, Kyoto 602-8566, Japan; 2 Department of Food Science, Ishikawa Prefectural University, 1-308 Suematsu, Nonoichi, Ishikawa 921-8836, Japan; 3 Nutrition Division, Taiyo Kagaku Co. Ltd., 1-3 Takaramachi, Yokkaichi, Mie 510-0844, Japan; 4 Laboratory of Animal Science, Department of Agricultural and Life Sciences, Kyoto Prefectural University, 1-5 Shimogamohangi-cho, Sakyo-ku, Kyoto 606-8522, Japan; 5 Gastroenterology, Tokyo Medical University Ibaraki Medical Center, 3-20-1 Ami-machi Chuo, Inashiki-gun, Ibaraki 300-0395, Japan

**Keywords:** *Akkermansia*, dysbiosis, epigallocatechin-3-gallate, high-fat diet, taurine-conjugated bile acids

## Abstract

Gut microbiota have profound effects on bile acid metabolism by promoting deconjugation, dehydrogenation, and dehydroxylation of primary bile acids in the distal small intestine and colon. High-fat diet-induced dysbiosis of gut microbiota and bile acid dysregulation may be involved in the pathology of steatosis in patients with non-alcoholic fatty liver disease. Epigallocatechin-3-gallate (EGCG), the most abundant polyphenolic catechin in green tea, has been widely investigated for its inhibitory or preventive effects against fatty liver. The aim of the present study was to investigate the effects of EGCG on the abundance of gut microbiota and the composition of serum bile acids in high-fat diet-fed mice and determine the specific bacterial genera that can improve the serum bile acid dysregulation associated with EGCG anti-hepatic steatosis action. Male C57BL/6N mice were fed with the control diet, high-fat diet, or high-fat diet + EGCG at a concentration of 0.32% for 8 weeks. EGCG significantly inhibited the increases in weight, the area of fatty lesions, and the triglyceride content in the liver induced by the high-fat diet. Principal coordinate analysis revealed significant differences in microbial structure among the groups. At the genus level, EGCG induced changes in the microbiota composition in high-fat diet-fed mice, showing a significantly higher abundance of *Adlercreutzia*, *Akkermansia*, *Allobaculum* and a significantly lower abundance of *Desulfovibrionaceae*. EGCG significantly reversed the decreased population of serum primary cholic acid and β-muricholic acid as well as the increased population of taurine-conjugated cholic acid, β-muricholic acid and deoxycholic acid in high-fat diet-fed mice. Finally, the correlation analysis between bile acid profiles and gut microbiota demonstrated the contribution of *Akkermansia* and *Desulfovibrionaceae* in the improvement of bile acid dysregulation in high-fat diet-fed mice by treatment with EGCG. In conclusion, the present study suggests that EGCG could alter bile acid metabolism, especially taurine deconjugation, and suppress fatty liver disease by improving the intestinal luminal environment.

## Introduction

Non-alcoholic fatty liver disease (NAFLD) has become an important public health issue because of its high prevalence.^(1)^ The spectrum of NAFLD ranges from simple hepatic steatosis, commonly associated with obesity, to non-alcoholic steatohepatitis, which can progress to fibrosis, cirrhosis, and hepatocellular carcinoma.^(2)^ Numerous factors are complexly involved in the pathology of NAFLD, including inflammatory factors such as lipopolysaccharides, cytokines, lipid overload, and genetic predisposition, but their mechanisms have not been clarified. Studies are gradually revealing the abnormalities of gut microbiota and their metabolites in patients with NAFLD,^(3,4)^ as well as in animal models induced by a high-fat diet (HFD).^(5)^ Hildebrandt *et al.*^(5)^ first reported dysbiosis characterized by decreases in Proteobacteria and Firmicutes and a decrease in the Bacteroidetes in HFD-fed mice. This result has since been reproduced in many experiments. Recent metagenomic analysis by next generation sequencing has demonstrated that the Proteobacteria phylum is closely involved in hepatic fibrosis in NAFLD patients^(6)^ and that *Bilophila wadsworthia*, a member of the Proteobacteria phylum, could aggravate an HFD-induced inflammatory response.^(7)^

In addition, when considering the mechanism by which dysbiosis of the resident microbiota could modify the pathology of NAFLD, several studies have revealed an important role of metabolites produced by microbiota using mass spectrometer (MS) analysis. Gut microbes synthesize many products such as short-chain fatty acids,^(8)^ trimethylamine,^(9)^ ammonia,^(10)^ and hydrogen.^(11)^ A recent study reported evidence of a link between hepatic steatosis/fibrosis and the gut microbiome-derived metabolite 3-(4-hydroxyphenyl) lactate, which is a product of aromatic amino acid metabolism,^(12)^ through a prospective cohort study. On the other hand, numerous human clinical trials of bile acids targeting gastrointestinal disease have been carried out.^(13–15)^ In recent years, several metabolites of bile acids (BAs) have been shown to regulate lipid and carbohydrate metabolism as well as energy homeostasis, in both hepatic and extrahepatic tissues, through positive and negative regulation of the activation of BA-specific receptors, farnesoid X receptor (FXR), and transmembrane G protein-coupled receptor (TGR)-5. The primary BAs synthesized in the human liver are cholic acid (CA) and chenodeoxycholic acid (CDCA). They are conjugated with glycine or taurine (Glyco-CA, Glyco-CDCA, Tauro-CA, and Tauro-CDCA), and then excreted into the bile. In the terminal ileum and the colon, bile salt hydrolase is expressed in various bacteria that deconjugate glycine and taurine. The hydroxyl group at the C-7α position of the deconjugated BAs is then dehydroxylated to form secondary BAs, deoxycholic acid (DCA), and lithocholic acid (LCA) by multi-step reactions of specific bacteria.^(16)^ In addition, hydroxyl groups at the C-3α, 7α, and 12α positions of both conjugated and unconjugated BAs can be dehydrogenated to carbonyl groups and further epimerized to 3β-, 7β-, and 12β-hydroxyl groups by intestinal bacteria. In mice, α-muricholic acid (MCA) and β-MCA are produced in the liver from CDCA as a primary BA, and these BAs were also conjugated by glycine and taurine and dehydroxylated to form ω-MCA as a secondary BA. A highly sensitive and specific method for the quantification of many conjugated/unconjugated primary and secondary BAs in serum and feces has been developed based upon a stable isotope dilution technique by liquid chromatography-tandem mass spectrometry (LC-MS/MS).^(16,17)^ However, it is poorly understood whether and to what extent gut microbiota are involved in the regulation of BAs and energy homeostasis, especially in the pathology of NAFLD.

Epigallocatechin-3-gallate (EGCG), the most abundant polyphenolic catechin in green tea, has been widely investigated in terms of its health benefits, including anti-inflammatory, anti-cancer, and anti-steatotic effects on the liver.^(18–23)^ Studies have demonstrated that treatment with EGCG suppressed fat deposition in the liver in high-fat diet (HFD)-fed mice, a murine model of human NAFLD, via the regulation of intracellular second messengers, signal transduction pathways, transcriptional activation, and autophagy pathway.^(24–27)^ In addition to these activities, the possibility that the functionality of EGCG could be derived from its influence on gut microbiota and intestinal environment has recently attracted attention, since catechins including EGCG have extremely low absorbability from the intestinal tract. It has been reported that EGCG reduced the abundance of *Clostridium* spp. and tends to increase the abundance of *Bacteroides* in rats.^(28)^ Recently, Sheng *et al.*^(29)^ reported that EGCG increases the abundance of *Akkermansia muciniphila* and promotes the release of glucagon-like peptide (GLP)-1 from the intestinal tract via the activation of TGR-5 in mice fed a Western diet.

To further investigate the concept that the gut microbiota-BA interaction may affect the development of NAFLD, we studied the influence of modulation of gut microbiota by EGCG on the serum composition of BAs and hepatic steatosis. Using a mouse model of NAFLD, we investigated the effects of EGCG on the abundance of gut microbiota by 16S rRNA sequencing and also determined the composition of serum BAs and the association between these two aspects. Finally, we determined the specific bacterial genera that improve the serum BA dysregulation associated with anti-hepatic steatosis after treatment with EGCG.

## Materials and Methods

### Animals

Five-week-old male C57BL/6N mice were obtained from Shimizu Laboratory Supplies (Kyoto, Japan). Mice were kept at 18–24°C and 40–70% relative humidity, with a 12 h light/dark cycle. They were allowed free access to water and food (CE-2; CLEA Japan, Tokyo, Japan) during a 1 week acclimatization period. Experimental procedures were conducted in accordance with the National Institutes of Health guidelines for the use of experimental animals. All experimental protocols were approved by the Animal Care Committee of Kyoto Prefectural University of Medicine (Permission #M27-474).

After acclimatization, the mice were divided into three groups of eight mice each. They were fed one of the following diets: control (CE-2; CLEA Japan), an HFD (HFD32; CLEA Japan), or an HFD with EGCG for 8 weeks. EGCG (Sunphenon^®^, Taiyo Kagaku Co., Ltd., Mie, Japan) was supplemented in the food at a concentration of 0.32%. In our study, the dose of EGCG used (0.32%) corresponded to consumption of approximately 10 cups of green tea per day in humans,^(30)^ based on an allometric scale.^(31)^ The CE-2 diet provided 32% protein, 59% carbohydrate, and 12% fat with a caloric value of 339 kcal/100 g. The HFD32 contained 25% protein, 29% carbohydrates, and 32% fat (with saturated, monounsaturated, and polyunsaturated fatty acids at 7, 22, and 4 g/100 g chow, respectively). HFD32 has a caloric value of 507 kcal/100 g. After 8 weeks of feeding, fresh stool samples were obtained from each mouse. Thereafter, the mice were sacrificed under anesthesia. Their blood, liver, epididymal adipose tissue, and cecal contents were collected immediately.

### Blood biochemical analysis

Blood supernatants were obtained as serum, after centrifugation at 1,500 × *g* for 10 min. Concentrations of triacylglyceride (TG), total cholesterol (T-Cho), non-esterified fatty acid (NEFA), aspartate amino-transferase (AST), alanine aminotransferase (ALT), high-density lipoprotein cholesterol (HDL-Cho) and low-density lipoprotein cholesterol (LDL-Cho) in serum were measured at SRL, Inc. (Tokyo, Japan).

### Histological analysis

The liver tissue samples were fixed in 20% formalin and embedded in paraffin. Sections (4 µm thick) were cut and stained with hematoxylin-eosin (HE). Histological features and the fat area in liver tissue sections were determined using a BZ-X710 fluorescence microscope (Keyence, Osaka, Japan). In each HE-stained section, the severity of hepatic histological steatosis was assessed and the ratio of fatty lesions was calculated as fatty area/(total area-vascular area) × 100 (%) using computer-assisted image analysis with All-in-One analysis software (KEYENCE SOFTWARE Co., Osaka, Japan), which were performed blind to prevent observer bias.

### Lipid analysis

TG concentration in the liver was measured enzymatically with a test kit (Triglyceride E Test Wako; Wako Pure Chemical Co. Ltd., Osaka, Japan). Lipids within the liver were extracted, using previously described methods.^(32)^

### Collection of cecal contents and extraction of bacterial genomic DNA

Fresh cecal contents were collected, placed into tubes, and kept at −80°C until further use. Bacterial genomic DNA was extracted from the cecal content samples, as previously described.^(33)^ Briefly, whole bacterial DNA was extracted from cecal content by using the QuickGene DNA Tissue kit SII (Kurabo, Osaka, Japan), with a nucleic acid extraction machine (QuickGene-Mini80; Kurabo).

### Library preparation and DNA sequencing

Preparation of the library for DNA sequencing was conducted as previously described^(34)^ using a MiSeq desktop sequencer (Illumina, San Diego, CA). Briefly, the V3–4 region of the 16S rRNA genes in each sample was amplified using the KAPA HiFi HotStart Ready Mix (Kapa Biosciences, Wilmington, MA), with primers 341F and 805R that contained a 5' overhang adapter sequence. The amplicon was purified by NucleoFast 96 PCR plates (Takara Bio Inc., Shiga, Japan). A second PCR was carried out using the KAPA HiFi HotStart Ready Mix to attach a unique combination of dual indices (I5 and I7 index) and Illumina sequencing adapters to each sample. The amplicon of the second PCR was purified, and the concentration was normalized with a SequalPrep Normalization Plate Kit (Life Technologies Japan, Tokyo, Japan). Each of the normalized amplicons was then evenly pooled and concentrated using AMPure XP beads (Beckman Coulter, Tokyo, Japan). From the library, 11 pM were combined with phiX Control (v3, Illumina; expected 20%) and sequenced using a 300 bp paired-end strategy on the MiSeq (Illumina), as per the manufacturer’s instructions.

### Sequence data analysis

Sequence data analysis was performed using USEARCH v8.0^(35)^ and QIIME v1.9.0^(36)^ as previously described.^(34)^ Statistical differences (*p*<0.05) in the relative abundance of bacterial phyla and genera between groups were evaluated using Student’s paired *t* test. The observed Chao1 and Shannon phylogenetic diversity indices were calculated using the R “phyloseq” package^(37)^ and were statistically analyzed using a Kruskal-Wallis test followed by the Steel-Dwass post hoc test. The β-diversity was estimated using the UniFrac metric to calculate distances between the samples and visualized by principal coordinate analysis (PCoA); it was statistically examined using permutational multivariate analysis of variance (PERMANOVA). The final figures were generated using QIIME (ver. 1.9.0).

### Determination of the serum BA profile

Serum BA profiles were detected using a liquid chromatography-tandem mass spectrometry (LC-MS/MS) system, as previously described.^(17,38)^ Briefly, 20 µl of mouse serum was diluted 100-fold with ^2^H-labelled internal standards and 0.5 M potassium phosphate buffer (pH 7.4). The mixture was injected into a Bond Elut C18 cartridge (200 mg; Agilent Technologies, Santa Clara, CA). Target molecules were eluted in water/ethanol (1:9, vol/vol). The eluate was evaporated under nitrogen until dry and then dissolved in 20 mM ammonium acetate buffer (pH 7.5)/methanol (1:1, vol/vol). An aliquot of the resulting sample solution was injected into the LC-MS/MS system for analysis. Chromatographic separation was performed using a Hypersil GOLD column (150 × 2.1 mm, 3 µm; Thermo Fisher Scientific, Waltham, MA). A mixture of 20 mM ammonium acetate buffer (pH 7.5), acetonitrile, and methanol (70:15:15, vol/vol/vol) was used for the initial mobile phase and was gradually changed to 30:35:35 (vol/vol/vol) over 30 min.

### Statistical analysis

All data are presented as mean ± SEM. Comparisons among the three groups were performed using one-way analysis of variance (ANOVA) with Tukey’s post hoc tests. Body weight gain curves were analyzed with an ANOVA, followed by Tukey’s multiple comparison tests. These statistical analyses were performed using statistical software (Graph Pad Prism, ver. 6.07; Graph Pad Software, San Diego, CA). The Pearson’s rank correlation coefficient was used to analyze the correlation between gut microbiota and BA. The correlation and outcome analysis was performed using the JMP statistical software package ver. 14. (SAS Institute, Inc., Cary, NC). All results were considered statistically significant at *p*<0.05.

## Results

### EGCG inhibited body weight increase and lipid accumulation in HFD-fed mice

**Fig. 1:**
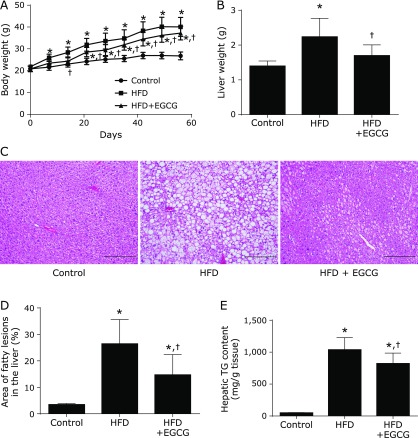
Inhibitory effect of EGCG on the HFD-induced obese phenotype and hepatic triacylglycerol accumulation. (A) Time-course of mouse body weight. Body weight of male mice receiving an EGCG-containing diet for 8 weeks. (B) Liver weight. (C) Hematoxylin-eosin (HE)-stained liver sections. (D) Area of fatty lesions in the liver. (E) Hepatic triacylglycerol (TG) content. C57BL/6N mice were fed a CE-2 diet (control), a high-fat diet (HFD), or an HFD supplemented with 0.32% EGCG (HFD + EGCG) for 8 weeks. Values are expressed as the means and SEM of eight mice in each group. Significant differences compared with the control group are denoted * (*p*<0.05), and those with the HFD group are denoted ^†^ (*p*<0.05). Photographs are of HE staining of liver sections from representative mice of each group (scale bar = 500 µm).

Six-week-old mice were fed for 8 weeks with either the control diet, HFD, or HFD supplemented with 0.32% EGCG. Body weight and food consumption were monitored weekly throughout the experiment. As shown in Fig. 1A, body weight was significantly higher in the HFD group than in the control group after 1 week of treatment (i.e., at 7 weeks of age). This difference was maintained through the end of the study. EGCG treatment significantly suppressed the body weight gain of mice during weeks 3–8 (Fig. 1A). During the experiment, the average food intake of mice in the HFD group was significantly lower than the control group. However, the food consumption of mice between the HFD group and the HFD + EGCG group was not different during the experiment (Supplemental Table 1*). The HFD-induced body weight gain was suppressed in the HFD + EGCG group. The final liver weight at the end of the experiment was significantly increased in the HFD group compared to the control group, and this increase was significantly inhibited by treatment with EGCG (Fig. 1B). Although the histological liver sections of the control group were free of lipid droplets, increased accumulation of lipid droplets was observed in the HFD group, leading to a condition of hepatic steatosis, and this increase was suppressed in the HFD + EGCG group (Fig. 1C). Consistent with the histological findings, the area of fatty lesions in the liver was significantly increased in the HFD group compared to the control, and this increase was significantly inhibited by treatment with EGCG (Fig. 1D). In addition, the content of TG in the liver was significantly increased in the HFD group, and this increase was also inhibited by treatment with EGCG (Fig. 1E).

The biochemical parameters of the serum obtained from mice at the end of the experiment are shown in Table 1. Serum levels of ALT and AST in the HFD group tended to increase compared to the control, and these increased levels tended to decrease in the HFD + EGCG group. However, these changes were not significant. Compared with the control, mice fed with the HFD exhibited significantly higher plasma NEFA, T-Cho, HDL-Cho, and LDL-Cho levels. EGCG supplementation had no effect on the HFD-mediated up-regulation of these parameters.

### Effects of EGCG on HFD-induced gut microbiota dysbiosis

**Fig. 2:**
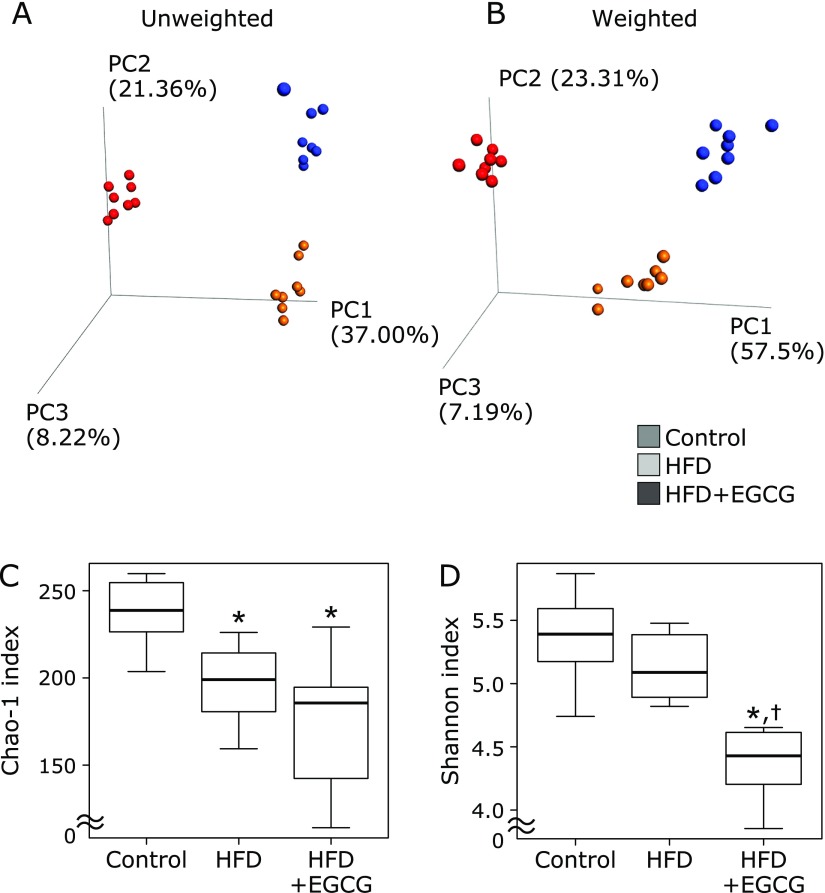
Effects of EGCG administration on gut microbiota in α and β diversity indices. (A) Principal coordinate analysis (PCoA) plot of unweighted UniFrac data. (B) PCoA plot of weighted UniFrac data. (C) The Chao 1 index (OTU richness estimation). (D) The Shannon index (OTU evenness estimation). C57BL/6N mice were fed with the control CE-2 diet (control), a high-fat diet (HFD), or the HFD supplemented with 0.32% EGCG (HFD + EGCG) for eight weeks. The composition of cecal content was analyzed using Illumina-based 16S rRNA sequencing. Values are expressed as the means and SEM of six mice in each group. Significant differences compared with the control group are denoted * (*p*<0.05), and those with the HFD group are denoted ^†^ (*p*<0.05).

In total, 412 OTUs were detected from Illumina high-quality sequence reads. Initially, the overall structure of the cecal microbiota among the control, HFD, and HFD + EGCG groups using β-diversity indices were calculated for unweighted and weighted UniFrac distances. PCoA revealed that there were significant microbial structural differences among the control, HFD and HFD + EGCG groups in unweighted (PERMANOVA, *p* = 0.0001) and weighted (PERMANOVA, *p* = 0.0001) UniFrac distances (Fig. 2A and B). Subsequently, we compared α-diversity among the control, HFD and HFD + EGCG groups using different indices the observed species and Chao 1 index (OTU richness estimation), and the Shannon index (OTU evenness estimation). The Chao 1 index was decreased in the HFD group compared to the control, and the decrease was not affected by EGCG (Fig. 2C). The Shannon index was significantly decreased in the HFD + EGCG group compared to the control, as well as to the HFD group (Fig. 2D).

**Fig. 3:**
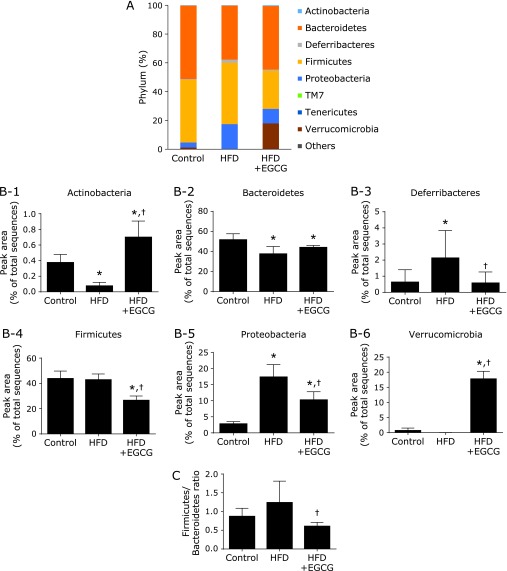
Effects of EGCG administration on gut microbiota at the phylum level. (A) The microbial composition at the phylum level. (B) At the phylum level of microbiota (calculated as the percentage of total microbiota). (C) Firmicutes/Bacteroidetes ratio. C57BL/6N mice were fed with the control CE-2 diet (control), a high-fat diet (HFD), or the HFD supplemented with 0.32% EGCG (HFD + EGCG) for eight weeks. The composition of cecal content was analyzed using Illumina-based 16S rRNA sequencing. Values are expressed as the means and SEM of six mice in each group. Significant differences compared with the control group are denoted * (*p*<0.05), and those with the HFD group are denoted ^†^ (*p*<0.05).

**Fig. 4:**
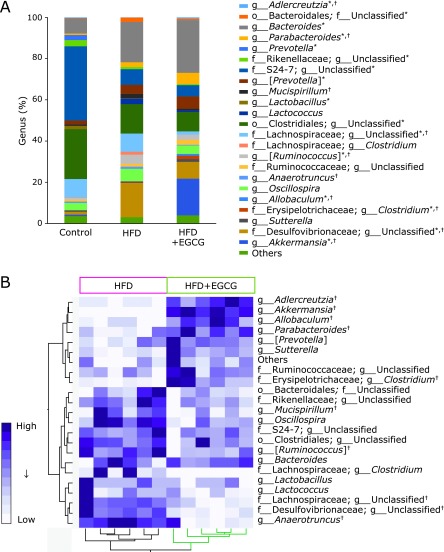
Effects of EGCG administration on gut microbiota at the genus level. (A) The microbial composition at the genus level. (B) Heat map of 16S rRNA gene sequencing analysis of cecal content at the genus level. The scale reflects the data as follows: violet indicates high values whereas white indicates low values for the percentage of reads that were classified at that rank. C57BL/6N mice were fed with the control CE-2 diet (control), a high-fat diet (HFD), or the HFD supplemented with 0.32% EGCG (HFD + EGCG) for eight weeks. The composition of cecal content was analyzed using Illumina-based 16S rRNA sequencing. Significant differences compared with the control group are denoted * (*p*<0.05), and those with the HFD group are denoted ^†^ (*p*<0.05).

**Fig. 5:**
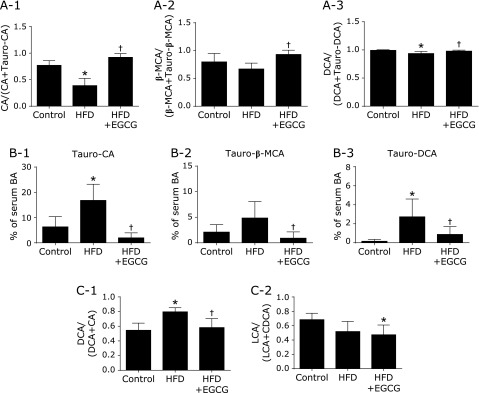
Determination of BA profile and calculation of BA transformation activities by gut microbiota. (A) Deconjugation. (B) Taurine-conjugated BAs. (C) 7α-Dehydroxylation. C57BL/6N mice were fed with the control CE-2 diet (control), a high-fat diet (HFD), or the HFD supplemented with 0.32% EGCG (HFD + EGCG) for eight weeks. Values are expressed as the means and SEM of six mice in each group. Significant differences compared with the control group are denoted * (*p*<0.05), and those with the HFD group are denoted ^†^ (*p*<0.05). CA, free cholic acid; Tauro-CA, taurine-conjugated cholic acid; β-MCA, free β-muricholic acid; Tauro-β-MCA, taurine-conjugated β-muricholic acid; DCA, free deoxycholic acid; Tauro-DCA, taurine-conjugated deoxycholic acid; BA, bile acid; LCA, free lithocholic acid; CDCA, free chenodeoxycholic acid.

**Fig. 6:**
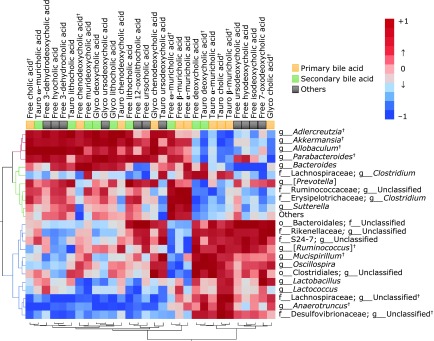
A heat map generated by Spearman’s correlation analysis reveals the relationship between the abundance of gut microbiota at the genus level and BA profile. Correlation heat map demonstrating the association between the indicated gut microbiota taxonomic genera and BAs. Red denotes a positive association, blue a negative association, and white no association. C57BL/6N mice were fed with a high-fat diet (HFD), or a HFD supplemented with 0.32% EGCG (HFD + EGCG) for eight weeks.

Compared to the control, HFD-fed mice had a lower abundance of phyla Actinobacteria and Bacteroidetes and had a higher abundance of phyla Deferribacteres and Proteobacteria (Fig. 3 and Supplemental Table 2*). Treating mice with EGCG induced the changes in microbiota composition in HFD-fed mice, showing a significantly higher abundance of the phyla Verrucomicrobia and Actinobacteria, and significantly lower abundance of the phyla Deferribacteres, Proteobacteria, and Firmicutes (Fig. 3B-1–6 and Supplemental Table 2*). The Firmicutes/Bacteroidetes ratio, an index of dysbiosis, was significantly decreased in the HFD + EGCG group compared to the HFD group (Fig. 3C).

Changes in gut microbiota induced by a HFD were also observed at the genus level (Fig. 4 and Table 2). Compared to the control, HFD-fed mice had a significantly lower abundance of 6 genera and had a significantly higher abundance of 8 genera shown in Fig. 4A and Table 2. Treating mice with EGCG induced the changes in microbiota composition in HFD-fed mice, showing significantly higher abundance of the genera *Adlercreutzia*, *Akkermansia*, *Allobaculum*, *Parabacteroides*, f_*Erysipelotrichaceae*; g_*Clostridium* which clustered together, and a significantly lower abundance of the genera *Mucispirillum*, [*Ruminococcus*], f*_Lachnospiraceae*; g_Unclassified, f_*Desulfovibrionaceae*; g_Unclassified, and *Anaerotruncus* which clustered together (Fig. 4A, B and Table 2).

### Effects of EGCG on serum levels of unconjugated/conjugated BAs

To investigate the effects of changes in gut microbiota on BA metabolism, we conducted a panel of BAs in the serum. A total of 17 unconjugated and 13 conjugated BAs, shown in Table 3, were quantified. Data from the serum BA profiles showed that HFD significantly decreased the level of total free BAs, and the reduction was significantly reversed by EGCG supplementation. Among free BAs, CA, ω-MCA, ursodeoxycholic acid (UDCA), ursocholic acid (UCA), hyodeoxycholic acid (HDCA), and 7-oxodeoxycholic acid (7-oxo DCA) were significantly decreased in the HFD group compared to the control group. The HFD-induced decrease in CA, a predominant primary BA in mice, was significantly reversed by treatment with EGCG. The level of free deoxycholic acid (DCA), a predominant secondary BA in mice, was significantly increased in the HFD group, but this increase was not affected by the EGCG treatment (Table 3).

In all 3 groups, glycine-conjugated BAs were almost below the detection limit in the mouse serum, but taurine-conjugated forms of primary and secondary BAs were detected. Contrary to the changes in free BAs, HFD increased the total level of taurine-conjugated BAs, and this increase was significantly inhibited by treatment with EGCG. Among taurine-conjugated BAs, Tauro-CA and Tauro-DCA were significantly increased in the HFD group compared to the control, and these increases were significantly inhibited by EGCG (Fig. 5B-1). The level of Tauro-β-MCA was also significantly decreased in the HFD + EGCG group compared to the HFD group (Fig. 5B-2).

To estimate the activities of BA transformation by gut microbiota and the effects of EGCG on transformation of BAs by deconjugation or 7α-dehydroxylation, we calculated the product/(product + substrate) ratio for each reaction. Deconjugation was calculated, and the CA/(CA + Tauro-CA) ratio and DCA/(DCA + Tauro-DCA) ratios were significantly decreased in the HFD group (Fig. 5A-1). These HFD-induced decreases in the deconjugation ratio were significantly reversed by treatment with EGCG. The β-MCA/(β-MCA + Tauro-β-MCA) ratio (β-MCA being a mouse-specific primary BA) was significantly increased in the HFD + EGCG group only (Fig. 5A-2). 7α-Dehydroxylation by gut microbiota was calculated by the DCA/(DCA + CA) ratio or LCA/(LCA + CDCA) ratio. The ratio of DCA/(DCA + CA) was significantly increased in the HFD group compared to the control, and this increase was significantly inhibited by treatment with EGCG (Fig. 5C-1). There was no significant difference in the LCA/(LCA + CDCA) ratio between the HFD and the HFD + EGCG group (Fig. 5C-2).

### Correlation between gut microbiota and BA composition

To clarify the relationship between gut microbiota (22 genera and others) and BAs (17 unconjugated and 13 conjugated BAs), we used Pearson’s correlation test (Fig. 6 and Supplemental Table 3*). The heat map data revealed a positive correlation between serum predominant primary CA and CDCA and *Adlercreutzia*, *Akkermansia*, or *Allobaculum* which clustered closely. A similar positive correlation was also seen between mouse-specific primary BAs (α-MCA and β-MCA) and f_*Erysipelotrichaceae*; g_*Clostridium*, or f_*Ruminococcaceae*; g_Unclassified, which clustered together. In addition, *Desulfovibrionaceae*; g_Unclassified, *Anaerotruncus*, and *Lachnospiraceae*; g_Unclassified*,* which clustered together, were correlated negatively and positively to the composition of CA and CDCA and to those of taurine-conjugated BAs (Tauro-CA, Tauro-α-MCA Tauro-β-MCA, or Tauro-DCA), respectively.

**Fig. 7:**
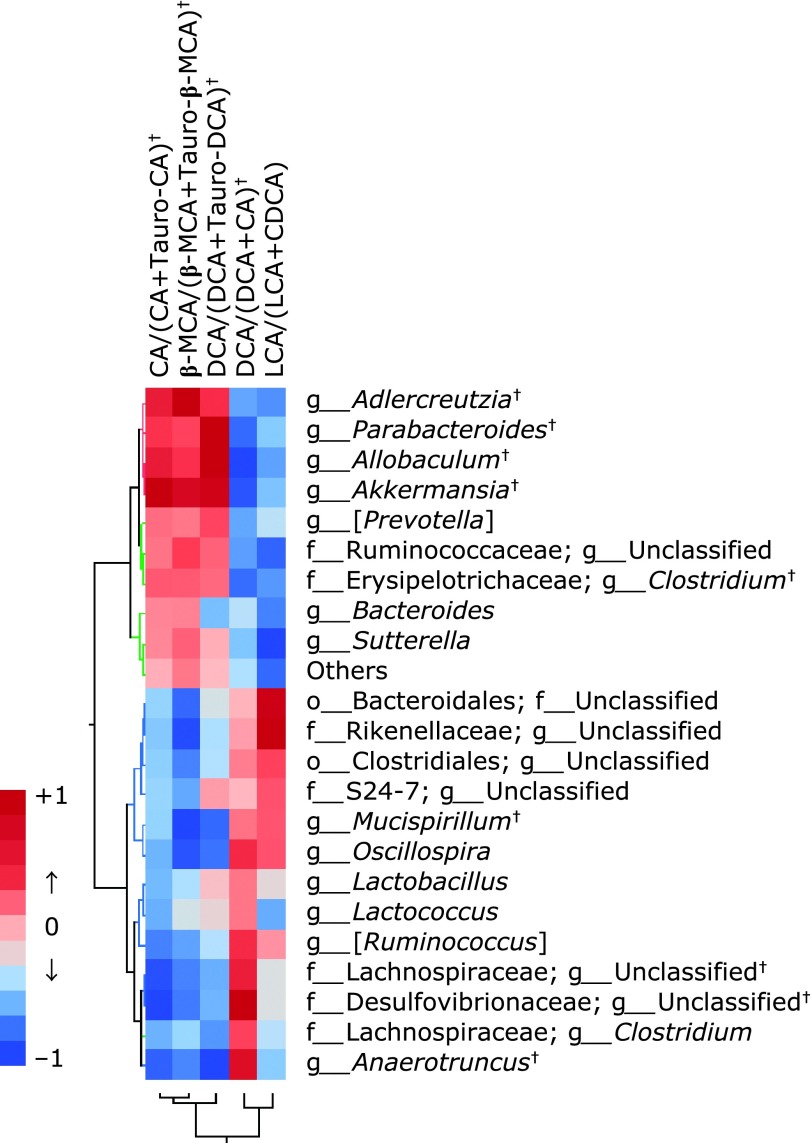
Correlation heat map demonstrating the association between the indicated gut microbiota taxonomic genera and BA composition. Red denotes a positive association, blue a negative association, and white no association. CA, free cholic acid; Tauro-CA, taurine-conjugated cholic acid; β-MCA, free β-muricholic acid; Tauro-β-MCA, taurine-conjugated β-muricholic acid; DCA, free deoxycholic acid; Tauro-DCA, taurine-conjugated deoxycholic acid; LCA, free lithocholic acid; CDCA, free chenodeoxycholic acid.

Finally, we investigate the correlation between BA transformation index and the abundance of gut microbiota. As shown in Fig. 7 and Supplemental Table 4*, significant positive correlations were observed between serum CA/(CA + Tauro-CA) and *Akkermansia*, *Allobaculum*, *Adlercreutzia*, or *Parabacteroides*, and between DCA/(DCA + CA) and f_*Desulfovibrionaceae*; g_Unclassified*, Anaerotruncus*, f_*Lachnospiraceae*; g_Unclassified, *or* [*Ruminococcus*].

## Discussion

In the present study, we investigated the effect of oral administration of EGCG on the gut microbiota and serum BA profile in mice fed a HFD by 16S rRNA sequencing analysis and quantitative systematic LC-MS/MS, respectively. We found EGCG significantly inhibited the increase in histological fatty deposit and TG accumulation in the liver induced by an HFD and also improved gut dysbiosis. In addition, EGCG significantly reversed the decrease in the serum level of primary BAs and the increases in those of secondary BAs and taurine-conjugated BAs in mice fed an HFD. Finally, we determined crucial genera of gut microbiota correlated with BA fluctuation in serum. These results demonstrate a novel anti-fatty liver action of EGCG, which is hardly absorbed from the intestinal tract, by modulation of BA metabolism to improve gut microbiota.

A number of findings indicate that dysbiosis of gut microbiota occurs in NAFLD in humans, and novel treatments targeting microbiota and their metabolites have recently been developed to address this issue.^(3,39)^ The mouse fatty liver model through HFD used in this study has been used previously^(40)^ and the abnormality of gut microbiota determined by 16sRNA metagenomic analysis has already been reported.^(5)^ In the present study, PCoA showing β-diversity clearly distinguished control, HFD and HFD + EGCG groups. At the phylum level, the relative abundance of Actinobacteria was significantly decreased and those of Deferribacteres and Proteobacteria were significantly increased in the HFD group as compared to the control group; EGCG significantly reversed these changes. The Firmicutes/Bacteroidetes ratio, an index of dysbiosis, tended to increase in the HFD group as compared to the control, and this increase was significantly inhibited in the HFD + EGCG group. These results suggest that EGCG improved dysbiosis of gut microbiota induced by HFD and, as a result, this improvement may be involved in the anti-fatty liver action of EGCG.

One key finding in this study is that the abundance of Proteobacteria phylum increased markedly in the HFD group compared with the control group, and this increase was significantly suppressed in the HFD + EGCG group. Recently, *Bilophila wadsworthia* belonging to p_Proteobacteria; f_*Desulfovibrionaceae*; g_*Bilophila* was identified as a bacterium exacerbating metabolic disorder caused by HFD, and it has been demonstrated that *Bilophila wadsworthia* bacteria aggravates the metabolic dysfunction in HFD-fed mice by enhancing intestinal mucosal permeability, promoting inflammatory immune response, and altering BA metabolism.^(7)^ Although g_*Bilophila* was hardly detected in this study, a related genus, p_Proteobacteria; f_*Desulfovibrionaceae*; g_Unclassified, increased more than 10-fold in the HFD group as compared with the control, and EGCG significantly inhibited this increase. These results suggest that f_*Desulfovibrionaceae* may play a crucial role in the development of HFD-induced fatty liver in this rodent model.

p_Actinobacteria contain important beneficial bacteria which comprise about 10% of the Japanese gut microbiota,^(41)^ and administration of *Bifidobacterium*, which is a major genus in this phylum, could ameliorate HFD-induced fatty liver in mice.^(42,43)^ In the present study, the abundance of p_Actinobacteria was significantly decreased in HFD-fed mice compared to control, and EGCG reversed this decrease and the levels increased more in the EGCG-treated group as compared to the control. However, in this experimental study, increased bacteria at Actinobacteria phylum by EGCG was f_*Coriobacteriaceae*; g_*Adlercreutzia*, but not g_*Bifidobacterium*. Some *Adlercreutzia* genera could metabolize and decompose EGCG into smaller molecular weight catechins (epicatechin, catechin, etc.), like *Adlercreutzia equolifaciens*.^(44,45)^ In the future, when considering the function of EGCG, it may be necessary to analyze microbiota that could metabolize or decompose EGCG into smaller molecule catechin metabolites.

In the present study, the most striking feature was the significant increase of the *Akkermansia* genus in the Verrucomicobia phylum in the HFD + EGCG group, consistent with recent reports.^(29,46)^ Recently, Sheng *et al.*^(29)^ reported that *Akkermansia* genus is significantly increased by western diet with EGCG administration. *Akkermansia muciniphila* is the main genus classified in the Verrucomicrobia phylum and recent studies revealed the involvement of this bacteria in obesity, sugar metabolism, and intestinal immunity.^(47)^ In previous studies, cranberry extracts,^(48)^ concord grape polyphenols,^(49)^ and apple procyanidin^(50)^ have been interestingly reported to show an anti-obesity effect through an increased abundance of the *Akkermansia* genus.^(51)^ In addition, these effects have also been reproduced by the administration of *Akkermansia muciniphila* to mice.^(52)^ Such polyphenols, including EGCG, generally have low absorption in the intestinal tract, and they reach the large intestine without being digested and absorbed, but the details of the mechanism by which the abundance of *Akkermansia* increases have not been elucidated. It is thought that *Akkermansia muciniphila* produces short-chain fatty acids such as acetic acid by feeding intestinal mucin and supplies energy to goblet cells that produce mucin. This study suggested that the marked increase in *Akkermansia* by EGCG treatment may be involved in hepatoprotection via various mechanisms. *Akkermansia* genus in feces may be one of the good surrogate markers to consider the functionality of EGCG. A human clinical trial is necessary to confirm the functionality of EGCG with respect to the gut microbiome.

In mice, CA, CDCA, α-MCA and β-MCA are primary BAs. In this study, free forms of these primary BAs were detected in the serum of the control mice, and the most predominant one was CA (about 60% of primary BAs). In the present study, the proportion of CA in serum markedly decreased in the HFD group and this decrease was significantly recovered to the equivalent levels of the control by the treatment with EGCG. The proportion of CDCA also increased about twice in the EGCG group as compared with the HFD group. CA and CDCA are ligands that bind most strongly to BA receptor FXR (CA<CDCA), and it is suspected that FXR activity was promoted by treatment with EGCG. Recently, Sheng *et al.*^(29)^ has reported that EGCG increased the concentration of FXR agonists, especially CDCA and CA, in serum and their regulated signaling-associated gene expression in the liver of mice, which supports our hypothesis. When FXR, a BA receptor present in the liver, is activated, SREBP1c is suppressed, TG synthesis is downregulated, and fatty liver is finally inhibited. These data suggest that EGCG increases serum levels of CA and CDCA through improving the intestinal environment, leading to the improvement of liver lipid metabolism and the inhibition of fatty liver. Furthermore, Tauro-CA increased markedly in the HFD group as compared with the control group, in contrast to the CA value, which markedly decreased in the HFD + EGCG group. In addition to Tauro-CA, the levels of other taurine-conjugated BAs (β-MCA, CDCA, and DCA) significantly increased in the HFD group as compared with the control, and these increases were markedly inhibited in the EGCG group. As Tauro-β-MCA is a natural antagonist of FXR,^(53)^ the reduction of Tauro-β-MCA also contributes to the activation of FXR in EGCG treated mice. Thus, the reduction of serum taurine-conjugated BAs by EGCG appears to be another important mechanism explaining the anti-fatty liver action of EGCG.

From the correlation between microbiota abundance obtained by 16S rRNA metagenomic analysis and serum BA profiles obtained by quantitative LC-MS/MS, we further examined the functionality of EGCG from the standpoint of the interaction of these markers. Judging from the distribution of correlation coefficients by heat map analysis, the abundance of *Akkermansia* and *Parabacteroides* genus showed a positive correlation with CA and a negative correlation with Tauro-CA, significantly increased in the EGCG group as compared with the HFD group and was 1.0% or more. As we suspected that Tauro-CA was recovered in the EGCG group due to the deconjugation reaction of Tauro-CA by intestinal bacteria with the bile salt hydrolase (*bsh*) gene, we searched for genera positively correlated with the ratio of CA/CA + Tauro-CA, β-MCA/β-MCA + Tauro-β-MCA, and DCA/DCA + Tauro-DCA, as indices of deconjugation of BAs. As a result, the abundance of *Akkermansia* and *Parabacteroides* genera showed significant and strong correlation with the deconjugation indices. The above results indicate that the increased abundance of *Akkermansia* and/or* Parabacteroides* may be involved in promoting the taurine deconjugation reaction from taurine-conjugated BAs to free type of BAs. However, the deconjugation activity^(54)^ of *Akkermansia* is not confirmed, and further investigation is required to confirm the same.

Since the production of secondary BA depends on the 7α-hydroxylation gene of gut microbiota, heat map analysis was also performed on the abundance of microbiota correlated with the ratio of DCA/DCA + CA and LCA/LCA + CDCA, as indices of 7α-hydroxylation of BAs. The present study showed that the ratio of DCA/DCA + CA was significantly increased in the HFD group compared with the control, and this increase was significantly inhibited by EGCG. Five genera were chosen as bacteria significantly correlated with the 7α-hydroxylation ratio; f_*Lachnospiraceae*; g_Unclassified, g_*[Ruminococcus]*, and g_*Oscillospira* with moderate positive correlation and g_*Anaerotruncus* and f_*Desulfovibrionaceae*; g_Unclassified with strong positive correlation. Among the five genera, f_*Desulfovibrionaceae*; g_Unclassified is high abundance in the HFD group, suggesting the possibility that this bacterium was key in the production of DCA, but it is unknown how much it contributes to secondary BA production.

The present study has several limitations. This study was based on serum BA and gut microbiota analysis, without information on BA concentrations in the liver and intestinal tract, changes in the intestinal BA transport system, or the expression of BA synthesis gene in the liver. More information will be required to improve our understanding of the gut-microbiome-liver axis. Furthermore, since we used homogeneous C57BL/6N mice fed an HFD as a model of NAFLD, there are issues in the application of these findings to human beings. As we focused on the role of taurine-conjugated BAs associated with the development of fatty liver, glycine-conjugated BAs, a predominant form in humans, should be analyzed in future studies on patients with NAFLD.

Through quantitative systematic LC-MS/MS we found that serum taurine-conjugated BAs (Tauro-CA, Tauro-β-MCA, and Tauro-DCA) increased in HFD-fed mice and that the correlation analysis between BA profiles and gut microbiota demonstrated the contribution of *Akkermansia* and f_*Desulfovibrionaceae*; g_Unclassified in the modulation of BA metabolism induced by EGCG treatment. The present study suggests that EGCG could alter BA metabolism and suppress fatty liver by improving the intestinal luminal environment.

## Author Contributions

YN and YI directed the paper’s conception. CU and YN wrote the paper. TT, KU, and YH provided intellectual support and modified the language. CU, KM, and YH performed the experiments. ZY and TO prepared EGCG. RI analyzed the sequence data. AH and YM analyzed the BA data. All authors read and approved the final manuscript for publication.

## Data Availability Statement

The datasets generated for this study can be found in the DDBJ Sequence Read Archive Database (DDBJ accession number; PRJDB7523).

## Funding

This work was supported by Grants-in-Aid for Scientific Research (B) (JSPS KAKENHI) to YN (Grant Number JP 16H05289) and by Grant of Industry-Academia-Government Collaboration of “Field for Knowledge Integration and Innovation” (FKII) to YN (No. 16824414) from the Ministry of Agriculture, Forestry and Fisheries of Japan.

## Figures and Tables

**Table 1:** Effect of HFD and EGCG on serum metabolic parameters at 8 weeks of treatment

	Control	HFD	HFD + EGCG
TG (mg/dl)	68 ± 6	35 ± 4*	25 ± 2*
NEFA (mEQ/L)	1,198 ± 119	1,514 ± 80*	1,250 ± 52
T-Cho (mg/dl)	82 ± 5	216 ± 8*	197 ± 9*
AST (IU/L)	67 ± 10	105 ± 21	72 ± 5
ALT (IU/L)	27 ± 3	48 ± 20	33 ± 5
HDL-Cho (mg/dl)	54 ± 3	80 ± 3*	70 ± 2*
LDL-Cho (mg/dl)	8 ± 1	29 ± 3*	30 ± 3*

C57BL/6N mice were fed with the control CE-2 diet (control), a High-fat diet (HFD), or the HFD supplemented with 0.32% EGCG (HFD + EGCG) for 8 weeks. Values are expressed as the means ± SEM of eight mice in each group; *p<0.05 compared with the control group. TG, triacylglycerol; NEFA, non-esterified fatty acid; mEQ, milliequivalent; T-Cho, total cholesterol; AST, aspartate amino-transferase; ALT, alanine amino-transferase; HDL-Cho, high-density lipoprotein cholesterol; LDL-Cho, low-density lipoprotein cholesterol.

**Table 2:** Average abundance of genus-level OTUs in the C57BL/6N mice treated with control, HFD, or HFD + EGCG

(%)							
Taxon					Control	HFD	HFD + EGCG
Phylum	Class	Order	Family	Genus			
Actinobacteria	Coriobacteriia	Coriobacteriales	Coriobacteriaceae	*Adlercreutzia*	0.36 ± 0.11	0.08 ± 0.05*	0.70 ± 0.21*^,†^
Bacteroidetes	Bacteroidia	Bacteroidales	Unclassified	Unclassified	0.11 ± 0.12	2.06 ± 1.70*	0.69 ± 0.33*
Bacteroidetes	Bacteroidia	Bacteroidales	Bacteroidaceae	*Bacteroides*	7.41 ± 2.54	19.68 ± 10.43*	25.44 ± 3.30*
Bacteroidetes	Bacteroidia	Bacteroidales	Porphyromonadaceae	*Parabacteroides*	0.60 ± 0.14	2.23 ± 2.46	5.58 ± 2.60*^,†^
Bacteroidetes	Bacteroidia	Bacteroidales	Prevotellaceae	*Prevotella*	2.49 ± 0.87	0.00 ± 0.00*	0.00 ± 0.00*
Bacteroidetes	Bacteroidia	Bacteroidales	Rikenellaceae	Unclassified	3.01 ± 0.45	1.08 ± 0.23*	0.55 ± 0.28*
Bacteroidetes	Bacteroidia	Bacteroidales	S24-7	Unclassified	35.96 ± 3.56	7.61 ± 1.95*	5.65 ± 2.03*
Bacteroidetes	Bacteroidia	Bacteroidales	[Paraprevotellaceae]	[*Prevotella*]	2.08 ± 1.46	4.34 ± 2.73	5.80 ± 2.06*
Deferribacteres	Deferribacteres	Deferribacterales	Deferribacteraceae	*Mucispirillum*	0.63 ± 0.78	2.13 ± 1.71*	0.58 ± 0.69^†^
Firmicutes	Bacilli	Lactobacillales	Lactobacillaceae	*Lactobacillus*	1.54 ± 1.24	0.16 ± 0.16*	0.08 ± 0.07*
Firmicutes	Bacilli	Lactobacillales	Streptococcaceae	*Lactococcus*	0.00 ± 0.00	2.66 ± 2.85*	0.84 ± 0.56
Firmicutes	Clostridia	Clostridiales	Unclassified	Unclassified	24.20 ± 0.89	14.28 ± 0.42*	9.49 ± 1.03*
Firmicutes	Clostridia	Clostridiales	Lachnospiraceae	Unclassified	8.98 ± 2.30	8.91 ± 3.02	1.55 ± 1.04*^,†^
Firmicutes	Clostridia	Clostridiales	Lachnospiraceae	*Clostridium*	0.23 ± 0.18	1.35 ± 1.86	0.01 ± 0.04
Firmicutes	Clostridia	Clostridiales	Lachnospiraceae	[*Ruminococcus*]	0.88 ± 0.42	4.51 ± 0.97*	2.35 ± 1.20*^,†^
Firmicutes	Clostridia	Clostridiales	Ruminococcaceae	Unclassified	0.93 ± 0.19	1.25 ± 0.65	2.50 ± 0.75
Firmicutes	Clostridia	Clostridiales	Ruminococcaceae	*Anaerotruncus*	0.55 ± 0.23	1.14 ± 0.29*	0.31 ± 0.38^†^
Firmicutes	Clostridia	Clostridiales	Ruminococcaceae	*Oscillospira*	3.68 ± 1.58	6.06 ± 1.42*	4.05 ± 1.92
Firmicutes	Erysipelotrichi	Erysipelotrichales	Erysipelotrichaceae	*Allobaculum*	0.00 ± 0.00	0.18 ± 0.21	0.91 ± 0.56*^,†^
Firmicutes	Erysipelotrichi	Erysipelotrichales	Erysipelotrichaceae	*Clostridium*	0.00 ± 0.00	0.26 ± 0.16	1.58 ± 1.46*^,†^
Proteobacteria	Betaproteobacteria	Burkholderiales	Alcaligenaceae	*Sutterella*	0.70 ± 0.33	0.40 ± 0.21	1.54 ± 1.63
Proteobacteria	Deltaproteobacteria	Desulfovibrionales	Desulfovibrionaceae	Unclassified	1.38 ± 0.51	16.48 ± 3.66*	8.11 ± 1.03*^,†^
Verrucomicrobia	Verrucomicrobiae	Verrucomicrobiales	Verrucomicrobiaceae	*Akkermansia*	0.66 ± 0.85	0.00 ± 0.04	17.76 ± 2.54*^,†^
				Others (<0.01)	3.65 ± 0.17	3.16 ± 0.10	3.95 ± 0.20

C57BL/6N mice were fed with the control CE-2 diet (control), a high-fat diet (HFD), or the HFD supplemented with 0.32% EGCG (HFD + EGCG) for 8 weeks. Values are expressed as the means ± SEM (%) of eight mice in each group; *p<0.05 compared with the control group. ^†^*p*<0.05 compared with the HFD group.

**Table 3:** Relative bile acid profiles of the serum

(%)				
		Control	HFD	HFD + EGCG
Free-	Cholic acid	19.66 ± 3.32	9.83 ± 2.46*	22.97 ± 5.45^†^
	ω-Muricholic acid	11.90 ± 4.29	3.08 ± 0.87*	13.44 ± 3.58^†^
	α-Muricholic acid	2.09 ± 0.75	0.91 ± 0.72	1.47 ± 1.03
	β-Muricholic acid	8.70 ± 2.73	10.19 ± 5.25	9.47 ± 5.76
	Chenodeoxycholic acid	2.49 ± 1.20	2.51 ± 0.71	4.35 ± 1.52*^,†^
	Deoxycholic acid	24.01 ± 5.82	38.83 ± 6.07*	33.16 ± 9.47
	Lithocholic acid	5.46 ± 2.44	2.92 ± 1.47	3.95 ± 1.40
	Ursodeoxycholic acid	3.17 ± 1.81	1.12 ± 0.64*	0.85 ± 0.50*
	Ursocholic acid	0.35 ± 0.17	0.08 ± 0.10*	0.09 ± 0.11*
	Hyocholic acid	0.03 ± 0.04	0.01 ± 0.02	0.09 ± 0.19
	Murideoxycholic acid	0.14 ± 0.11	0.33 ± 0.14	0.42 ± 0.20*
	Hyodeoxycholic acid	2.45 ± 0.60	1.18 ± 0.75*	1.14 ± 0.33*
	Isodeoxycholic acid	1.65 ± 0.71	1.12 ± 0.88	0.84 ± 0.23
	3-Dehydrocholic acid	0.13 ± 0.11	0.03 ± 0.03	0.21 ± 0.25
	7-Oxodeoxycholic acid	4.60 ± 1.85	0.87 ± 0.57*	1.00 ± 0.46*
	3-Dehydrodeoxycholic acid	0.03 ± 0.03	0.08 ± 0.06	0.16 ± 0.15
	12-Oxolithocholic acid	1.52 ± 0.88	0.80 ± 0.50	1.01 ± 0.34
	
	Free-bile acid	88.38 ± 7.12	73.90 ± 3.10*	94.62 ± 2.10^†^

Glyco-	Cholic acid	0.09 ± 0.04	0.30 ± 0.65	0.04 ± 0.04
	Chenodeoxycholic acid	0.08 ± 0.06	0.02 ± 0.02	0.05 ± 0.07
	Deoxycholic acid	0.02 ± 0.02	0.01 ± 0.01	0.15 ± 0.20
	Lithocholic acid	0.00 ± 0.00	0.00 ± 0.00	0.01 ± 0.01
	Ursodeoxycholic acid	0.02 ± 0.04	0.00 ± 0.00	0.01 ± 0.02
	
	Glyco-bile acid	0.22 ± 0.09	0.32 ± 0.64	0.27 ± 0.26

Tauro-	Cholic acid	6.33 ± 4.05	16.80 ± 6.42*	1.97 ± 1.98^†^
	ω-Muricholic acid	2.09 ± 2.04	0.59 ± 0.62	0.64 ± 0.43
	α-Muricholic acid	0.59 ± 0.72	0.55 ± 0.52	0.34 ± 0.62
	β-Muricholic acid	2.09 ± 1.47	4.84 ± 3.23	0.90 ± 1.24^†^
	Chenodeoxycholic acid	0.05 ± 0.04	0.13 ± 0.05	0.20 ± 0.20
	Deoxycholic acid	0.16 ± 0.20	2.73 ± 1.88*	0.87 ± 0.83^†^
	Lithocholic acid	0.03 ± 0.03	0.03 ± 0.04	0.04 ± 0.06
	Ursodeoxycholic acid	0.08 ± 0.08	0.11 ± 0.14	0.13 ± 0.17
	
	Tauro-bile acid	11.41 ± 7.05	25.78 ± 3.28*	5.11 ± 2.16^†^

C57BL/6N mice were fed with the control CE-2 diet (control), a high-fat diet (HFD), or the HFD supplemented with 0.32% EGCG (HFD + EGCG) for 8 weeks. Values are expressed as the means ± SEM (%) of six mice in each group; *p<0.05 compared with the Control group. ^†^*p*<0.05 compared with the HFD group.

## Abbreviations

BA: bile acid
CA: cholic acid
CDCA: chenodeoxycholic acid
DCA: deoxycholic acid
EGCG: epigallocatechin-3-gallate
FXR: farnesoid X receptor
HFD: high fat diet
LCA: lithocholic acid
MCA: muricholic acid
NAFLD: non-alcoholic fatty liver disease
PCoA: principal coordinate analysis
Tauro-β-MCA: taurine-conjugated β-muricholic acid
Tauro-CA: taurine-conjugated cholic acid
Tauro-DCA: taurine-conjugated deoxycholic acid
TG: triacylglyceride
