# Supplementary material for: Green tea polyphenol (epigallocatechin-3-gallate) improves gut dysbiosis and serum bile acids dysregulation in high-fat diet-fed mice
Source: J Clin Biochem Nutr. 2019 Apr 6;65(1):34–46. doi: 10.3164/jcbn.18-116 (PMC6667385; doi:10.3164/jcbn.18-116)
Supplement: Supplemental Table 1 [file jcbn18-116st01.pdf]

Supplemental Table 1. Body weight and organ weight of mice

	Control	HFD	HFD + EGCG
Initial body weight (g)	20.85 ± 0.42	21.83 ± 0.27	20.82 ± 0.35
Final body weight (g)	26.76 ± 0.57	40.07 ± 1.37*	37.24 ± 0.95*,†
Body weight gain (g)	5.91 ± 0.41	18.24 ± 1.17*	16.42 ± 0.78*
Food intake (g/day)	2.30 ± 0.05	1.69 ± 0.07*	1.58 ± 0.05*
Liver weight (g)	1.39 ± 0.05	2.23 ± 0.17*	1.69 ± 0.09†
Liver/body weight ratio (%)	5.22 ± 0.19	5.52 ± 0.27	4.54 ± 0.20†
Epididymal adipose tissue (g)	0.44 ± 0.04	2.07 ± 0.13*	2.05 ± 0.08*
Epididymal adipose tissue/body weight ratio (%)	1.63 ± 0.12	5.14 ± 0.28*	5.50 ± 0.15*
Content of cecum (g)	0.57 ± 0.03	0.26 ± 0.01*	0.32 ± 0.05*
Content of cecum/body weight ratio (%)	2.12 ± 0.09	0.66 ± 0.04*	0.85 ± 0.13*

C57BL/6N mice were fed with the control CE-2 diet (control), a high-fat diet (HFD), or the HFD supplemented with 0.32% EGCG (HFD + EGCG) for 8 weeks. Values are expressed as the means ± SEM (%) of eight mice in each group; **p*<0.05 compared with the control group. †*p*<0.05 compared with the HFD group.
