# Supplementary material for: Green tea polyphenol (epigallocatechin-3-gallate) improves gut dysbiosis and serum bile acids dysregulation in high-fat diet-fed mice
Source: J Clin Biochem Nutr. 2019 Apr 6;65(1):34–46. doi: 10.3164/jcbn.18-116 (PMC6667385; doi:10.3164/jcbn.18-116)
Supplement: Supplemental Table 2 [file jcbn18-116st02.pdf]

**Supplemental Table 2.** Average abundance of phylum-level OTUs in C57BL/6N mice treated with control, HFD or HFD + EGCG

(%)

| Taxon (Phylum level) | Control | HFD | HFD + EGCG |
|---|---|---|---|
| Actinobacteria | 0.39 ± 0.11 | 0.08 ± 0.05* | 0.70 ± 0.21*,† |
| Bacteroidetes | 50.76 ± 6.34 | 37.58 ± 7.46* | 44.06 ± 2.10* |
| Deferribacteres | 0.67 ± 0.74 | 2.13 ± 1.71* | 0.57 ± 0.69† |
| Firmicutes | 43.61 ± 5.63 | 42.81 ± 4.65 | 26.55 ± 3.47*,† |
| Proteobacteria | 3.38 ± 1.93 | 17.36 ± 3.90* | 10.23 ± 2.56*,† |
| TM7 | 0.02 ± 0.07 | 0.00 ± 0.00 | 0.08 ± 0.17 |
| Tenericutes | 0.03 ± 0.07 | 0.00 ± 0.00 | 0.00 ± 0.00 |
| Verrucumicrobia | 1.10 ± 1.53 | 0.01 ± 0.04 | 17.76 ± 2.54*,† |
| Others | 0.00 ± 0.00 | 0.04 ± 0.05 | 0.05 ± 0.08 |

C57BL/6N mice were fed with the control CE-2 diet (control), a high-fat diet (HFD), or the HFD supplemented with 0.32% EGCG (HFD + EGCG) for 8 weeks. Values are expressed as the means ± SEM (%) of eight mice in each group; *$p<0.05$ compared with the control group. †$p<0.05$ compared with the HFD group.
