# Supplementary material for: Green tea polyphenol (epigallocatechin-3-gallate) improves gut dysbiosis and serum bile acids dysregulation in high-fat diet-fed mice
Source: J Clin Biochem Nutr. 2019 Apr 6;65(1):34–46. doi: 10.3164/jcbn.18-116 (PMC6667385; doi:10.3164/jcbn.18-116)
Supplement: Supplemental Table 3 [file jcbn18-116st03.pdf]

Supplemental Table 3. Correlation between bile acid and gut microbiota in HFD-fed mice

Taxon	Free-																
Spearman correlation	Cholic acid	ω-Muricholic acid	α-Muricholic acid	β-Muricholic acid	Chenodeoxycholic acid	Deoxycholic acid	Lithocholic acid	Ursodeoxycholic acid	Ursocholic acid	Hyocholic acid	Murideoxycholic acid	Hyodeoxycholic acid	Isodeoxycholic acid	3-Dehydrocholic acid	7-Oxodeoxycholic acid	3-Dehydrodeoxycholic acid	12-Oxolithocholic acid
g__Adlercreutzia	0.67*	0.55	−0.03	−0.03	0.18	−0.17	0.38	−0.26	0.07	0.35	0.37	−0.18	−0.35	0.49	−0.03	0.42	0.00
o__Bacteroidales; f__Unclassified	−0.15	−0.43	−0.38	−0.46	−0.21	0.25	0.40	0.62*	0.45	−0.18	−0.15	0.71*	0.81*	−0.20	0.51	−0.15	0.62*
g__Bacteroides	0.13	0.26	0.45	−0.05	0.38	−0.09	−0.08	−0.64*	−0.26	0.20	0.47	−0.55	−0.53	0.25	−0.51	−0.13	−0.33
g__Parabacteroides	0.63*	0.50	0.07	0.35	0.15	−0.47	0.46	0.25	0.36	0.29	0.23	0.32	0.09	0.38	−0.15	0.42	0.48
f__Rikenellaceae; g__Unclassified	−0.21	−0.68*	−0.32	−0.29	−0.10	0.39	0.05	0.39	0.26	−0.27	−0.11	0.43	0.47	−0.31	0.57	−0.27	0.32
f__S24-7; g__Unclassified	−0.04	−0.69*	−0.59*	−0.28	−0.06	0.47	0.01	0.40	0.29	−0.26	−0.10	0.66*	0.53	−0.28	0.58*	0.00	0.25
g__[Prevotella]	0.38	0.78*	0.08	0.16	−0.20	−0.66*	0.58*	0.24	0.20	0.15	−0.24	−0.08	0.22	0.25	0.20	0.10	0.36
g__Mucispirillum	−0.30	−0.50	0.03	−0.58*	−0.05	0.48	−0.05	0.13	−0.10	−0.21	0.08	0.15	0.15	−0.31	0.13	−0.31	0.16
g__Lactobacillus	−0.33	−0.26	−0.45	0.05	−0.31	0.22	−0.34	0.07	−0.01	−0.13	−0.54	−0.12	0.02	−0.15	0.62*	0.06	−0.34
g__Lactococcus	−0.44	−0.15	−0.27	0.28	−0.27	0.08	−0.50	0.03	−0.11	−0.09	−0.53	−0.10	−0.05	−0.15	0.18	0.14	−0.41
o__Clostridiales; g__Unclassified	−0.10	−0.83*	−0.52	−0.51	0.06	0.67*	−0.09	0.08	−0.04	−0.22	−0.16	0.26	0.17	−0.30	0.51	−0.05	−0.12
f__Lachnospiraceae; g__Unclassified	−0.79*	−0.38	−0.10	0.14	−0.32	0.17	−0.60*	0.16	−0.17	−0.37	−0.48	−0.06	0.14	−0.46	0.18	−0.31	−0.30
f__Lachnospiraceae; *g__Clostridium*	−0.51	−0.34	0.29	−0.12	−0.29	0.50	−0.45	−0.28	−0.34	−0.11	0.20	−0.13	−0.27	−0.25	−0.50	−0.26	−0.24
g__[Ruminococcus]	−0.53	−0.77*	−0.59*	−0.27	−0.02	0.59*	−0.20	0.32	0.22	−0.25	−0.10	0.60*	0.44	−0.30	0.25	0.01	−0.04
f__Ruminococcaceae; g__Unclassified	0.27	0.73*	0.60*	0.78*	−0.22	−0.63*	0.02	−0.08	−0.02	0.18	0.14	−0.30	−0.31	0.22	−0.45	0.01	0.16
g__Anaerotruncus	−0.85*	−0.13	0.13	−0.08	−0.25	0.06	−0.54	−0.02	−0.30	−0.31	−0.38	−0.21	0.13	−0.40	−0.12	−0.41	−0.33
g__Oscillospira	−0.42	−0.68*	−0.29	−0.76*	0.08	0.64*	−0.02	0.03	0.00	−0.17	0.02	0.21	0.26	−0.24	0.24	−0.24	−0.04
g__Allobaculum	0.95*	0.30	0.02	0.20	0.42	−0.37	0.26	−0.40	0.23	0.55	0.05	−0.15	−0.21	0.61*	0.05	0.49	0.03
f__Erysipelotrichaceae; *g__Clostridium*	0.38	0.86*	0.63*	0.73*	−0.17	−0.87*	0.22	0.10	−0.02	0.14	−0.04	−0.26	−0.13	0.17	−0.37	−0.03	0.32
g__Sutterella	0.09	0.98*	0.51	0.48	−0.31	−0.75*	0.14	0.06	−0.15	0.07	−0.14	−0.39	−0.14	0.12	−0.29	−0.04	0.11
f__Desulfovibrionaceae; g__Unclassified	−0.78*	−0.52	−0.31	−0.09	−0.24	0.36	−0.53	0.21	−0.12	−0.35	−0.43	0.18	0.26	−0.45	0.16	−0.16	−0.28
g__Akkermansia	0.78*	0.66*	0.23	0.30	0.17	−0.49	0.51	−0.08	0.15	0.36	0.30	−0.10	−0.25	0.47	−0.18	0.33	0.27
Others	−0.10	0.85*	0.29	0.40	−0.35	−0.61*	0.12	0.19	−0.03	0.09	−0.24	−0.28	−0.07	0.14	−0.13	0.08	0.09

Taxon	Glyco-				
Spearman correlation	Cholic acid	Chenodeoxycholic acid	Deoxycholic acid	Lithocholic acid	Ursodeoxycholic acid
g__Adlercreutzia	−0.21	0.28	0.52	0.53	0.42
o__Bacteroidales; f__Unclassified	0.54	0.15	−0.29	−0.24	−0.28
g__Bacteroides	−0.03	−0.10	0.30	0.26	0.25
g__Parabacteroides	−0.37	0.20	0.49	0.38	0.59*
f__Rikenellaceae; g__Unclassified	0.59*	−0.10	−0.39	−0.37	−0.31
f__S24-7; g__Unclassified	0.20	0.01	−0.27	−0.27	−0.19
g__[Prevotella]	0.25	0.24	0.00	0.12	−0.10
g__Mucispirillum	0.17	0.06	−0.32	−0.25	−0.34
g__Lactobacillus	0.35	−0.23	−0.39	−0.37	−0.45
g__Lactococcus	−0.09	−0.27	−0.25	−0.30	−0.25
o__Clostridiales; g__Unclassified	0.07	0.36	−0.21	0.05	−0.31
f__Lachnospiraceae; g__Unclassified	0.26	−0.54	−0.55	−0.65*	−0.47
f__Lachnospiraceae; *g__Clostridium*	−0.15	−0.21	−0.24	−0.31	−0.18
g__[Ruminococcus]	0.11	−0.12	0.01	−0.09	0.05
f__Ruminococcaceae; g__Unclassified	−0.11	−0.33	0.02	−0.17	0.22
g__Anaerotruncus	0.21	−0.49	−0.55	−0.63*	−0.55
g__Oscillospira	0.34	0.15	−0.13	−0.02	−0.26
g__Allobaculum	−0.27	0.38	0.24	0.37	0.18
f__Erysipelotrichaceae; *g__Clostridium*	−0.18	−0.08	−0.03	−0.07	0.11
g__Sutterella	−0.07	−0.17	−0.08	−0.14	−0.05
f__Desulfovibrionaceae; g__Unclassified	0.05	−0.32	−0.38	−0.45	−0.36
g__Akkermansia	−0.27	0.33	0.49	0.52	0.49
Others	0.02	−0.19	0.03	−0.08	0.02

Taxon	Tauro-							
Spearman correlation	Cholic acid	ω-Muricholic acid	α-Muricholic acid	β-Muricholic acid	Chenodeoxycholic acid	Deoxycholic acid	Lithocholic acid	Ursodeoxycholic acid
g__Adlercreutzia	−0.84*	0.45	−0.38	−0.79*	0.44	−0.54	0.55	0.30
o__Bacteroidales; f__Unclassified	0.32	−0.07	0.53	0.46	−0.13	0.05	−0.19	0.15
g__Bacteroides	−0.25	−0.10	−0.31	−0.54	0.13	0.32	0.14	0.07
g__Parabacteroides	−0.72*	0.45	−0.29	−0.47	0.27	−0.63*	−0.03	−0.03
f__Rikenellaceae; g__Unclassified	0.45	0.08	0.41	0.65*	−0.12	0.31	−0.28	0.18
f__S24-7; g__Unclassified	0.37	0.24	0.35	0.40	0.02	−0.01	−0.10	0.33
g__[Prevotella]	−0.55	0.07	−0.05	−0.27	−0.13	−0.66*	0.40	−0.20
g__Mucispirillum	0.36	−0.17	0.57	0.37	−0.16	0.69*	−0.14	0.40
g__Lactobacillus	0.40	0.02	−0.03	0.51	−0.06	−0.10	0.03	−0.05
g__Lactococcus	0.45	−0.16	−0.14	0.42	−0.09	−0.10	−0.18	−0.28
o__Clostridiales; g__Unclassified	0.47	0.12	0.66*	0.34	0.00	0.35	0.16	0.20
f__Lachnospiraceae; g__Unclassified	0.82*	−0.47	0.07	0.77*	−0.31	0.36	−0.45	−0.28
f__Lachnospiraceae; *g__Clostridium*	0.39	−0.31	−0.13	0.32	−0.20	0.48	−0.17	0.26
g__[Ruminococcus]	0.73*	−0.26	0.25	0.43	0.21	0.23	−0.45	−0.12
f__Ruminococcaceae; g__Unclassified	−0.58*	0.21	−0.77*	−0.12	−0.15	−0.50	−0.07	−0.11
g__Anaerotruncus	0.78*	−0.76*	0.15	0.50	−0.43	0.54	−0.33	−0.20
g__Oscillospira	0.59*	−0.33	0.61*	0.27	0.03	0.68*	−0.09	0.17
g__Allobaculum	−0.70*	0.78*	−0.26	−0.61*	0.04	−0.51	0.45	0.26
f__Erysipelotrichaceae; *g__Clostridium*	−0.63*	0.15	−0.40	−0.16	−0.31	−0.52	0.03	−0.27
g__Sutterella	−0.49	−0.17	−0.38	−0.21	−0.24	−0.46	0.16	−0.23
f__Desulfovibrionaceae; g__Unclassified	0.87*	−0.50	0.25	0.62*	−0.16	0.37	−0.40	−0.21
g__Akkermansia	−0.92*	0.51	−0.35	−0.65*	0.24	−0.62*	0.36	0.02
Others	−0.33	−0.25	−0.35	−0.08	−0.05	−0.43	−0.02	−0.35

JMP statistical software was used to calculate Pearson's correlation coefficient (*r*). C57BL/6N mice were fed with a high-fat diet (HFD), or the HFD supplemented with 0.32% EGCG (HFD + EGCG) for 8 weeks. Values are expressed six mice in each group; *$p<0.05$.
