# Supplementary material for: Green tea polyphenol (epigallocatechin-3-gallate) improves gut dysbiosis and serum bile acids dysregulation in high-fat diet-fed mice
Source: J Clin Biochem Nutr. 2019 Apr 6;65(1):34–46. doi: 10.3164/jcbn.18-116 (PMC6667385; doi:10.3164/jcbn.18-116)
Supplement: Supplemental Table 4 [file jcbn18-116st04.pdf]

Supplemental Table 4. Correlation between indices of deconjugation or 7α-dehydroxylation in serum and gut microbiota in HFD-fed mice

Taxon	Deconjugation			7α-Dehydroxylation	
Spearman correlation	CA/ (CA + Tauro-CA)	β-MCA/ (β-MCA + Tauro-β-MCA)	DCA/ (DCA + Tauro-DCA)	DCA/ (DCA + CA)	LCA/ (LCA + CDCA)
g__Adlercreutzia	0.81*	0.88*	0.51	−0.52	−0.29
o__Bacteroidales; f__Unclassified	−0.36	−0.71*	0.00	0.19	0.68*
g__Bacteroides	0.37	0.37	−0.35	−0.10	−0.32
g__Parabacteroides	0.73*	0.63*	0.62*	−0.74*	−0.16
f__Rikenellaceae; g__Unclassified	−0.43	−0.79*	−0.14	0.26	0.69*
f__S24-7; g__Unclassified	−0.35	−0.51	0.21	0.17	0.46
g__[Prevotella]	0.48	0.41	0.45	−0.52	−0.04
g__Mucispirillum	−0.37	−0.80*	−0.62*	0.41	0.45
g__Lactobacillus	−0.51	−0.21	0.11	0.40	0.06
g__Lactococcus	−0.56	−0.07	0.05	0.40	−0.24
o__Clostridiales; g__Unclassified	−0.39	−0.62*	−0.12	0.37	0.51
f__Lachnospiraceae; g__Unclassified	−0.88*	−0.62*	−0.41	0.66*	0.03
f__Lachnospiraceae; *g__Clostridium*	−0.54	−0.29	−0.49	0.57	−0.04
g__[Ruminococcus]	−0.72*	−0.53	−0.11	0.63*	0.27
f__Ruminococcaceae; g__Unclassified	0.45	0.66*	0.37	−0.55	−0.37
g__Anaerotruncus	−0.83*	−0.61*	−0.72*	0.71*	−0.15
g__Oscillospira	−0.54	−0.77*	−0.59*	0.63*	0.46
g__Allobaculum	0.82*	0.70*	0.62*	−0.86*	−0.26
f__Erysipelotrichaceae; *g__Clostridium*	0.57	0.53	0.35	−0.72*	−0.28
g__Sutterella	0.35	0.50	0.15	−0.37	−0.42
f__Desulfovibrionaceae; g__Unclassified	−0.91*	−0.64*	−0.39	0.76*	0.03
g__Akkermansia	0.94*	0.84*	0.60*	−0.82*	−0.19
Others	0.16	0.41	0.12	−0.14	−0.36

JMP statistical software was used to calculate Pearson's correlation coefficient (*r*). C57BL/6N mice were fed with a high-fat diet (HFD), or the HFD supplemented with 0.32% EGCG (HFD + EGCG) for 8 weeks. Values are expressed six mice in each group; $*p<0.05$.
